# Infrared Spectroelectrochemistry of Iron-Nitrosyl Triarylcorroles. Implications for Ligand Noninnocence

**DOI:** 10.1021/acs.inorgchem.9b03613

**Published:** 2020-02-13

**Authors:** Md. Hafizur Rahman, Michael D. Ryan, Hugo Vazquez-Lima, Abraham Alemayehu, Abhik Ghosh

**Affiliations:** † Department of Chemistry, Marquette University, 1414 West Clybourn Street, Milwaukee, Wisconsin 53233, United States; ‡ Department of Chemistry, UiT—The Arctic University of Tromsø, 9037 Tromsø, Norway; § Centro de Química, Instituto de Ciencias, Universidad Autónoma de Puebla, Edif. IC9, CU, San Manuel, 72570 Puebla, Puebla, Mexico

## Abstract

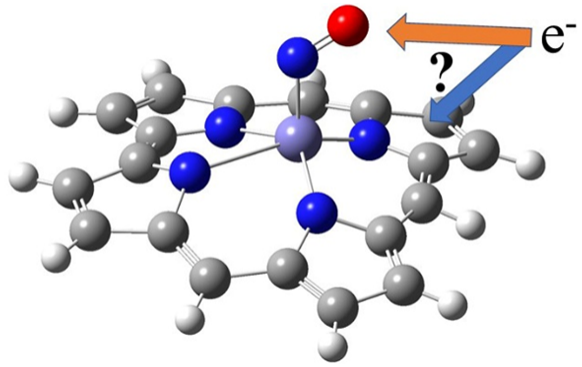

Recent DFT calculations have suggested that iron nitrosyl triarylcorrole complexes have substantial {FeNO}^7^–corrole^•2–^ character. With this formulation, reduction of Fe(C)(NO) complexes, where C = triarylcorrole, should be centered on the corrole macrocycle rather than on the {FeNO}^7^ moiety. To verify this proposition, visible and infrared spectroelectrochemical studies of Fe(C)(NO) were carried out and the results were interpreted using DFT (B3LYP/STO-TZP) calculations. The first reduction of Fe(C)(NO) led to significant changes in the Soret and Q-band regions of the visible spectrum as well as to a significant downshift in the ν_NO_ and changes in the corrole vibrational frequencies. DFT calculations, which showed that the electron was mostly added to the corrole ligand (85%), were also able to predict the observed shifts in the ν_NO_ and corrole bands upon reduction. These results underscore the importance of monitoring both the corrole and nitrosyl vibrations in ascertaining the site of reduction. By contrast, the visible spectroelectrochemistry of the second reduction revealed only minor changes in the Soret band upon reduction, consistent with the reduction of the FeNO moiety.

## Abstract 2

For the reduction of FeNO moiety or corrole, infrared spectroelectrochemistry and DFT calculations were performed and experimental evidence was obtained for the noninnocence of the corrole in Fe(triphenylcorrole)(NO).

## Introduction

For much of their 25-year history, iron–corrole–NO complexes have been regarded as unusually stable {FeNO}^6^ species, where the superscript numeral refers to the Enemark–Feltham electron count, i.e., the combined number of transition metal d and NO π* electrons.^1^ Recently, based on optical spectroscopy, broken-symmetry DFT calculations, and single-crystal X-ray structure determinations, an alternative formulation has been suggested for these complexes, namely, one involving substantial {FeNO}^7^–corrole^•2–^ character.^2^ The linearity of the FeNO unit is then explained as a result of antiferromagnetic coupling between the d_*z*^2^_ electron of the {FeNO}^7^ center of the porphyrin-a_2u_-like corrole radical.^3^ With such a formulation, the site of reduction for the Fe(corrole)(NO) complex should be at the corrole ligand rather than at the nitrosyl group. To verify this hypothesis, a combination of visible and infrared spectroelectrochemistry and DFT calculations were used to characterize the [Fe(corrole)(NO)]^−^ complex.

Iron–corrole nitrosyl complexes can be reversibly oxidized and reduced. For example, the Fe(OEC)(NO) complex, where OEC = octaethylcorrole, was reduced in cyclic voltammetry in two widely separated one-electron reversible waves (−0.41 and −1.92 V vs SCE).^4^ Two reversible oxidation waves were also observed.^4^ Similar voltammetric behavior has also been observed for other corrole derivatives.^2,5−7^ Autret et al. interpreted the first reduction wave to be due to the reduction of a ferric complex to a ferrous complex.^4^ The visible spectroelectrochemical reduction led to a shift in the Soret band from 376 to 412 nm and of the band at 536 to 577 nm, with a small band at 538 nm. A much smaller shift was observed in the Soret band by Singh et al. for the FeNO complex of tris(4-nitrophenyl)corrole.^6^ The visible spectroelectrochemistry of the tris(4-nitrophenyl) complex showed a red-shift of the Soret band from 382 to 395 nm, while in the longer wavelength region the 544 nm band split into two bands at 524 and 662 nm. Nardis et al.^5^ observed strongly split Soret bands in the UV–visible spectra of β-nitrocorrole derivatives which were significantly different from the unsubstituted derivatives.

The infrared spectroelectrochemistry of iron corrole nitrosyl complexes, focusing on the nitrosyl region from 1600 to 1900 cm^–1^, was first reported by Autret et al.^4^ The nitrosyl band for the Fe(OEC)(NO) complex downshifted by 182 cm^–1^ upon reduction. This shift was similar to the downshift for the Fe^III/II^(OEP)(NO)^0/–^ reduction (187 cm^–1^).^8^ Nardis et al.^5^ observed a downshift of 167 cm^–1^ for β-nitrocorrole derivatives.

The downshift in the ν_NO_ band does not necessarily indicate that the site of reduction is either on the iron or on the nitrosyl. Bonding between Fe and NO is complex, and the energy of the ν_NO_ also depends upon the geometry of the Fe–NO moiety.^9,10^ For iron porphyrin nitrosyls, the {FeNO}^6^ complexes exhibit nearly linear Fe–N–O units,^11^ while the analogous {FeNO}^7^ complexes exhibit distinctly bent ones (with Fe–N–O angles around 150°).^11,12^ The ν_NO_ band also downshifts from around 1854 cm^–1^ to around 1667 cm^–1^ upon reduction of the {FeNO}^6^ unit to {FeNO}^7^. Further reduction to {FeNO}^8^ leads to additional bending of the Fe–N–O moiety (down to about 127°),^13^ while the ν_NO_ band downshifts from about 1667 to 1440 cm^–1^.^13^ DFT calculations indicated that this reduction is centered on the nitrosyl group.^13^ For the formally {FeNO}^6^ corroles, the site of reduction is complicated by the noninnocence of the corrole ligand and the recent formulation of the Fe(corrole)NO as an {FeNO}^7^–corrole^•2–^ assembly.^3^ We hypothesized that characterization of the anionic, reduced derivatives, including ring vibrations, should provide additional evidence for this novel electronic–structural formulation where the corrole was predicted to be a noninnocent ligand.^3^ The primary focus of this work, accordingly, is on the changes in the nitrosyl and core vibrational frequencies of Fe(corrole)NO upon reduction and their correlation to DFT results. Consistency of the experimental spectra with the DFT results would provide further support for the noninnocence of the corrole in the neutral complex.

In addition to the infrared spectroelectrochemistry of the first reduction of Fe(corrole)(NO), the second reduction was also studied using visible spectroelectrochemistry (formation of {FeNO}^8^–corrole complexes). For Fe(porphyrin)(NO) complexes, relatively small changes were observed upon reduction to the {FeNO}^8^ complexes in the UV/visible spectra. While there have been voltammetric studies of the second reduction of Fe(corrole)(NO), the spectroelectrochemistry of these complexes has not been previously reported.

## Experimental Section

### Materials

Iron(II) chloride tetrahydrate [FeCl_2_·(4H_2_O)], tetrabutylammonium perchlorate (TBAP), sodium nitrite NaNO_2_, and tetrahydrofuran (THF) were purchased from Sigma-Aldrich Chemical Co. Deuterated tetrahydrofuran (THF-*d*_8_) and isotopic Na^15^NO_2_ were obtained from Cambridge Isotope Laboratories. Tetrahydrofuran was stirred for 1 day with sodium metal and benzophenone under argon. The solution was refluxed until it was a persistent dark blue color and was then collected under argon in a rubber-sealed bottle and stored in a glovebox. The TBAP was dried at 90 °C under vacuum overnight and stored in the glovebox before use. Iron–nitrosyl 5,10,15-*meso*-tris(4-trifluoromethylphenyl)corrole, Fe(T*p*CF_3_PC)(NO), iron–nitrosyl 5,10,15-*meso*-triphenylcorrole, Fe(TPC)(NO), iron–nitrosyl 5,10,15-*meso*-tris(4-methylphenyl)corrole, Fe(T*p*CH_3_PC)(NO), iron–nitrosyl 5,10,15-*meso*-tris(4-methyloxylphenyl)corrole, Fe(T*p*OCH_3_PC)(NO), and their ^15^NO isotopomers were synthesized according to previously established literature methods.^2^ All the complexes were found to be pure using visible spectroscopy and cyclic voltammetry.^14^

### Instrumentation

All the electrochemical and spectroelectrochemical experiments were performed using a potentiostat (model CHI 650D, CH Instruments). For all the voltammetric experiments, a platinum wire was used as the counter electrode and Ag/AgNO_3_ in acetonitrile was used as the reference electrode. The boron-doped diamond (BDD) electrode (3 mm diameter) was obtained from Windsor Scientific, Ltd. (Slough, U.K.) and was used as the working electrode, except as noted. For UV–visible spectroelectrochemical experiments, a low-volume thin-layer quartz glass cell was purchased from BAS Inc. The cell consists of three electrodes, i.e., a platinum mesh as a working electrode, a platinum wire as a counter electrode, and Ag/AgNO_3_ in acetonitrile as a reference electrode. The UV–visible spectra were collected using a HP 8452A diode array spectrophotometer. The FTIR spectroelectrochemical cell was built manually according to a method previously published in the literature.^15^ In the FTIR cell, a thin gold strip was used as the counter electrode and a silver wire as a pseudo-reference electrode. All the FTIR spectra were obtained using 64 scans and 2 cm^–1^ resolution on a Thermo Nicolet-FTIR spectrophotometer (model 670 Nexus) with an MCT detector cooled under liquid nitrogen.

### Procedures

All the electrochemical experiments were performed in a glovebox under a nitrogen atmosphere. For UV–visible and FTIR spectroelectrochemical experiments, the sample was prepared in the glovebox with its container sealed with Teflon tape and then with parafilm. The FTIR spectroelectrochemical experiments were carried out under a nitrogen atmosphere to ensure the absence of moisture. For both experiments (UV–visible and FTIR), a slow cyclic scan (1–5 mV/s) was applied to ensure complete electrolysis of the complexes at each potential. The potential was scanned to sufficiently negative potential to ensure complete electrolysis. All experiments were carried out in THF (THF-*d*_8_ for infrared spectroelectrochemistry) and 0.10 M TBAP.

### Calculations

All DFT calculations, including geometry optimizations and vibrational analyses, were carried out with the ADF 2016 program^16^ using the B3LYP^17^ functional, fine integration grids, and tight convergence criteria. The version of ADF that was used in this work led to minor differences in the calculated infrared spectrum for Fe(TPC)(NO) relative to an earlier work.^3^ Scalar relativistic effects were taken into account with the ZORA^18^ approximation and ZORA STO-TZP basis sets.^19^ Dispersion corrections were introduced via Grimme’s D3^20^ scheme.

## Results and Discussion

### Cyclic Voltammetry of Iron Corrole Nitrosyls

The cyclic voltammetric reduction of Fe(TPC)(NO) occurs in two well-separated waves at −0.53 and −1.86 V vs Ag/AgNO_3_ (Figure S1). The results for other corroles are summarized in Table 1 and were consistent with those from previous work, taking into account changes due to solvent and reference electrodes.^2,4,5^ The effect of substituents on the phenyl groups was rather small and generally paralleled known substituent effects. The Δ*E*_p_ values were slightly larger than theoretical values (about 100 mV versus 59 mV) where the difference is probably due to some residual uncompensated resistance. Much larger Δ*E*_p_ values were observed when the boron-doped diamond electrode was used and may be due to the semiconductor nature of the electrode.

**Table 1:** Cyclic Voltammetry of Iron Corrole Nitrosyls^c^

compound	reference electrode, solvent	*E*°_1_, V	*E*°_2_, V	ref
Fe(T*p*CF_3_PC)(NO)	Ag/AgNO_3_, THF^a^	–0.46	–1.99	this work
	SCE, CH_2_Cl_2_	–0.22	–	(2)
Fe(TPC)(NO)	Ag/AgNO_3_, THF	–0.53	–1.86	this work
	Fc^+^/Fc, CH_2_Cl_2_	–0.85	–	(7)
	SCE, CH_2_Cl_2_	–0.33	–	(2)
Fe(T*p*CH_3_PC)(NO)	Ag/AgNO_3_, THF	–0.50	–1.99	this work
	SCE, CH_2_Cl_2_	–0.36	–	(2)
	Fc^+^/Fc, CH_2_Cl_2_	–0.85	–	(7)
Fe(T*p*CH_3_OPC)(NO)	Ag/AgNO_3_, THF	–0.52	–1.97	this work
	SCE, CH_2_Cl_2_	–0.35	–1.74 irr	(5)
	SCE, CH_2_Cl_2_	–0.37	–	(2)
	Fc^+^/Fc, CH_2_Cl_2_	–0.87	–	(7)
Fe(OEC)(NO)	SCE, PhCN	–0.41	–1.92	(4)
	Fc^+^/Fc, CH_2_Cl_2_	–0.86	–	(7)
Fe(TNPC)(NO)^b^	Fc^+^/Fc, CH_2_Cl_2_	–0.63	–	(7)

a Working electrode: platinum.

b 5,10,15-tris(4-nitrophenyl)corrolate.

c Data for this work were obtained at 100 mV/s in THF with 0.10 M TBAP.

### Visible Spectroelectrochemistry of Fe(corrole)NO Complexes

**Figure 1:**
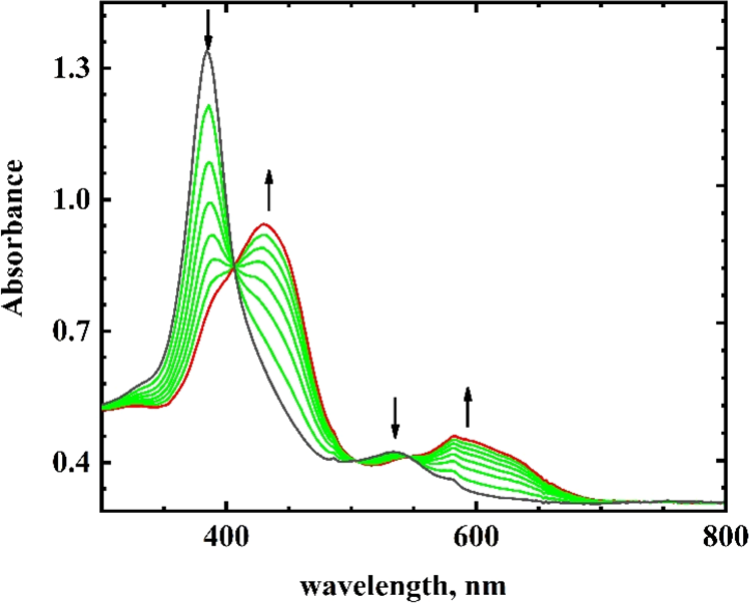
Visible spectroelectrochemistry of the first reduction of 0.3 mM Fe(T*p*CF_3_PC)(NO) in THF. Initial spectrum: −0.200 V (black); intermediate spectra: −0.551, −0.593, −0.620, −0.680, −0.730, −0.800 V (green); final spectrum: −0.701 V reverse scan (red). Electrolyte: 0.10 M TBAP.

The UV–visible spectroelectrochemistry of the first wave of Fe(T*p*CF_3_PC)(NO) is shown in Figure 1. The Soret band at 384 nm and the band at 534 nm disappeared upon reduction, and new bands at 430 and 582 nm appeared. Returning the potential to the initial potential led to the complete regeneration of the starting bands. The changes in the Soret band for Fe(T*p*CF_3_PC)(NO) were quite similar to the changes observed for Fe(OEC)(NO)^4^ (OEC = octaethylcorrole). In that case, the spectral changes were previously interpreted as due to reduction of the metal (Fe) center from the ferric to ferrous state.^4^ The observed changes in the Soret band and longer wavelength though were different from β-nitro and 4-nitrophenyl corrole complexes of FeNO^5,6^ (Table 2). Reduction of Fe(TPC)(NO) and Fe(T*p*CH_3_PC)(NO) resulted in similar changes (Figures S2 and S3). The spectral data are summarized in Table 2.

**Table 2:** UV/Visible Spectra of Iron Corrole Nitrosyl Complexes

compound	solvent	Soret, nm		Reference
Fe(TPC)(NO)	THF	386	532	this work
Fe(TPC)(NO)^−^	THF	418	576	this work
Fe(TPC)(NO)^2–^	THF	418	582	this work
Fe(T*p*CH_3_PC)(NO)	THF	398	534	this work
Fe(T*p*CH_3_PC)(NO)^−^	THF	412	582	this work
Fe(T*p*CH_3_PC)(NO)^2–^	THF	420	582, 700 sh	this work
Fe(T*p*CF_3_PC)(NO)	THF	384	534	this work
Fe(T*p*CF_3_PC)(NO)^−^	THF	430	582	this work
Fe(T*p*CF_3_PC)(NO)^2–^	THF	424	582	this work
Fe(T*p*O_2_N-PC)(NO)	CH_2_Cl_2_	382	544	(6)
Fe(T*p*O_2_N-PC)(NO)^−^	CH_2_Cl_2_	395	524, 662	(6)
Fe(NO_2_(TMOPC))(NO)^a^	CH_2_Cl_2_	359, 431	565	(5)
Fe(NO_2_(TMOPC))(NO)^−^a	CH_2_Cl_2_	391, 475	618, 728	(5)
Fe(OEC)(NO)	benzonitrile	376	536	(4)
Fe(OEC)(NO)^−^	benzonitrile	412	538, 577	(4)

a NO_2_(TMOPC) = 3-nitro-tris(4-methoxyphenyl)corrole

**Figure 2:**
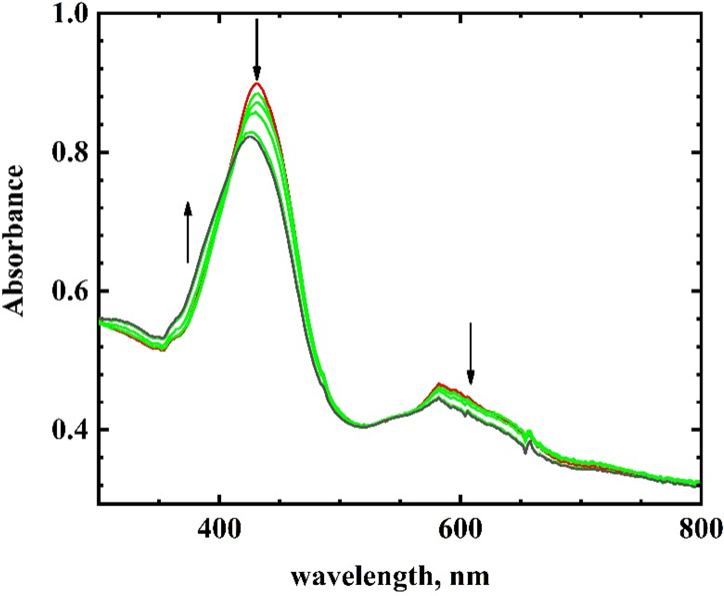
Visible spectroelectrochemistry for the second reduction of 0.3 mM Fe(T*p*CF_3_PC)(NO) in THF. Initial spectrum: −1.706 V (black); intermediate spectra: −1.736 V, −1.916 V, −1.934 V*, and −1.844 V* (green); final spectrum: −1.754 V* (red). *Potential from reverse scan. Electrolyte: 0.10 M TBAP.

Further reduction at the second wave yielded less significant changes in the UV–visible spectrum (Figure 2). The Soret band for Fe(T*p*CF_3_PC)(NO)^2–^ shifted to 424 nm in the dianion, as compared to the 430 nm band in the anion. There was a decrease in the molar absorptivity of this band upon reduction. In the long wavelength region, the molar absorptivity also decreased with minimal shifts on the band. The spectra for the other two complexes are shown in Figures S4 and S5, and the data are summarized in Table 2. The dianion complexes were stable in THF and the starting materials were regenerated by returning the electrode potential to the initial potential.

### Infrared Spectroelectrochemistry of the First Reduction of Fe–Corrole–NO Complexes

**Figure 3:**
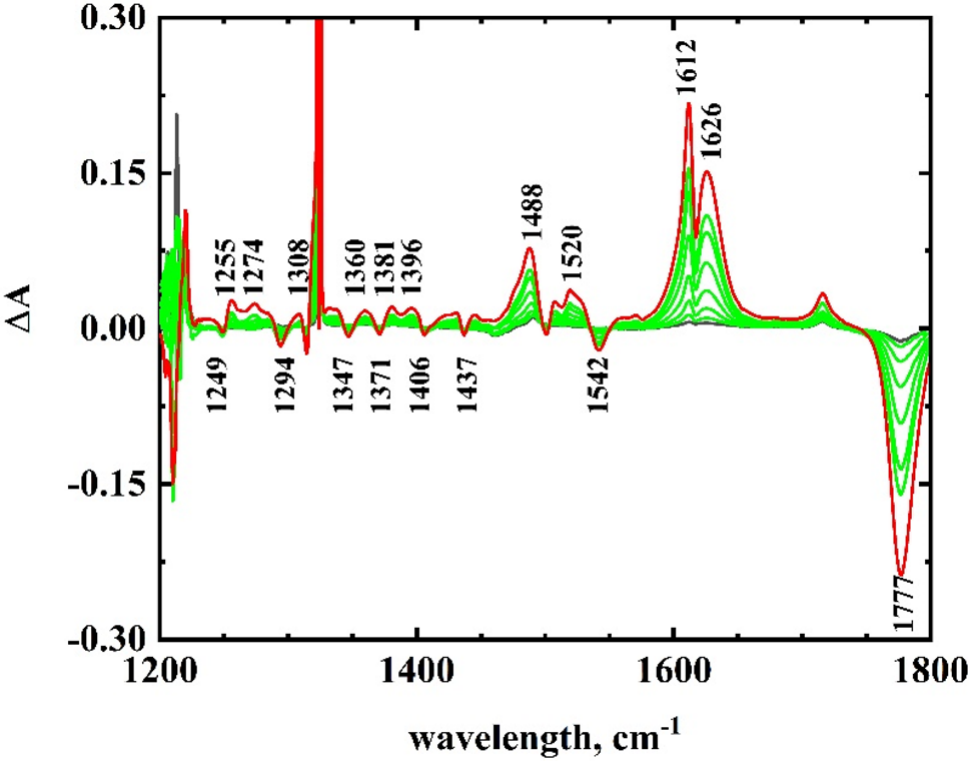
Infrared spectroelectrochemical difference spectra for the first reduction of 4.5 mM Fe(T*p*CF_3_PC)(NO) in THF-*d*_8_. Initial spectrum (black); intermediate spectra (green); final spectrum (red). Electrolyte: 0.10 M TBAP.

The reduction of Fe(T*p*CF_3_PC)(NO) was carried out in THF-*d*_8_ in order to have the widest spectral window. The difference spectra are shown in Figure 3. The nitrosyl band at 1777 cm^–1^ disappeared upon reduction, and two new bands appeared at 1612 and 1626 cm^–1^. Via the repetition of the infrared spectroelectrochemical experiment using the ^15^N isotopomer instead of natural abundance nitrogen (Figure S6), the ν_NO_ of Fe(T*p*CF_3_PC)(NO) shifted from 1777 to 1741 cm^–1^, while that of Fe(T*p*CF_3_PC)(NO)^−^ shifted from 1626 to 1596 cm^–1^. The 1612 cm^–1^ band remained essentially unchanged. The 1612 cm^–1^ band is a corrole vibration that shifted slightly upon reduction but also increased in its molar absorptivity. A comparison of Figure 3 with Figure S6 showed that the other vibrations were unchanged upon ^15^N isotopic substitution of the nitrosyl.

The downshift in the ν_NO_ band of 151 cm^–1^ upon reduction was similar to, but slightly smaller than, that observed in other studies of the reduction of iron–corrole–nitrosyl complexes. Autret et al.^4^ observed a somewhat larger downshift of 182 cm^–1^ for Fe(OEC)(NO) (OEC = octaethylcorrole), while Nardis et al.^5^ observed a 167 cm^–1^ downshift for the Fe(3-nitro-T*p*CH_3_OPC)(NO), both in methylene chloride. A comparison of the difference spectrum (Figure 3 or Figure S6) with the KBr spectrum in Figure S7 showed that most of the corrole vibrations also changed upon reduction. Using a solvent/electrolyte background spectrum, the infrared spectrum for Fe(T*p*CF_3_PC)(^15^NO) could be obtained in THF (Figure S8). Most of the bands proved much the same as in KBr, but there were some small shifts. The most significant shifts were a downshift in the ν_NO_ band from 1746 to 1741 cm^–1^ and in the 1618 cm^–1^ band to 1616 cm^–1^. The other negative bands, due to the disappearance of the starting corrole bands at 1542, 1437, 1406, 1371, 1347, 1294, and 1249 cm^–1^, can be observed in the KBr spectrum. The shifts in the nitrosyl band for all the complexes studied, as well as literature values, are summarized in Table 3.

**Table 3:** Infrared Spectroelectrochemistry of Iron Corrole Nitrosyls

compound	solvent	FeNO ν_NO_ (ν_15NO_) (cm^–1^)	FeNO^–^ ν_NO_ (ν_15NO_) (cm^–1^)	ref
Fe(TPC)(NO)	THF	1773 (1735)	1623 (1597)	this work
	DFT calcd	1794 (1758)	1650 (1623)	this work
Fe(T*p*CH_3_PC)(NO)	THF	1769 (1734)	1618 (1595)	this work
Fe(T*p*CH_3_OPC)(NO)	THF	1767	1620	this work
Fe(T*p*CF_3_PC)(NO)	THF	1777 (1741)	1626 (1596)	this work
Fe(OEC)(NO)	benzonitrile	1767	1585	(4)
(3-NO_2_T*p*CH_3_OPC)Fe(NO)	CH_2_Cl_2_	1786	1619	(5)
Fe(T*p*O_2_N-PC)(NO)	KBr	1775 (1733)	–	(6)

The spectrum for Fe(T*p*CF_3_PC)(^15^NO)^−^ is shown in Figure S9, after subtraction of the solvent/electrolyte, and residual amounts of starting material were subtracted from the solvent-subtracted Fe(T*p*CF_3_PC)(^15^NO)^−^ spectrum. Comparison of the two spectra showed that reduction of the starting complex led to downshifts in the corrole bands at 1616 cm^–1^ (to 1611 cm^–1^) and the 1542 cm^–1^ band to 1528 cm^–1^. The interpretation of these changes will be delayed until after the DFT calculations for Fe(TPC)(NO) and Fe(TPC)(NO)^−^ are presented.

### DFT Calculations

**Figure 4:**
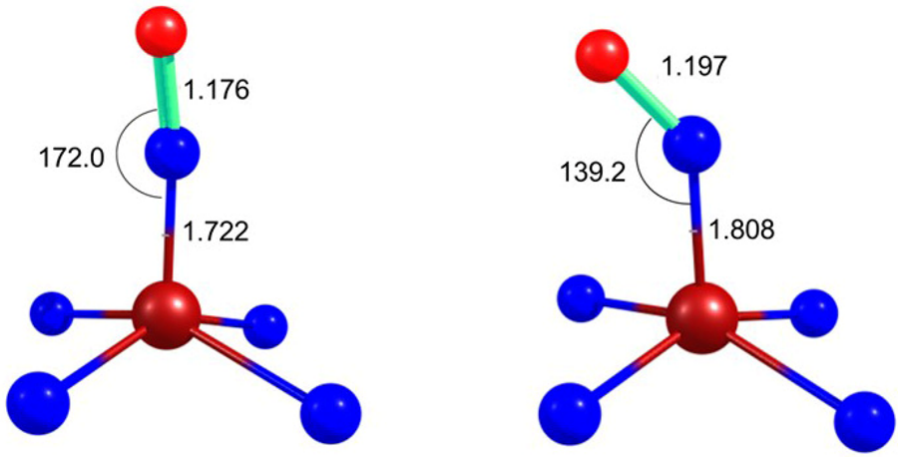
Central core of Fe(TPC)(NO) (left) and Fe(TPC)(NO)^−^ (right). Distances are shown in angstroms (Å) and angles in degrees.

All-electron spin-unrestricted (broken-symmetry) DFT (B3LYP/STO-TZP) calculations do a good job of reproducing the ν_NO_ of Fe(TPC)(NO) and Fe(TPC)(NO)^−^, with the calculated values being 1793.5 and 1650.1 cm^–1^, respectively, corresponding to a downshift of 143.4 cm^–1^. This downshift reflects both a lengthening of the calculated NO bond length from 1.176 to 1.197 Å upon reduction as well as significantly enhanced bending of the FeNO unit, from 170.0° in the neutral complex to 139.2° in the anion (Figure 4). The bending of the FeNO unit is similar to the FeNO angle in porphyrin complexes where the angle for {FeNO}^6^ shifts from ∼180° to ∼150° in {FeNO}^7^.^11,12^ Both the calculated frequencies and the downshift are in excellent accord with experimental values of 1773 and 1623 cm^–1^, respectively, with a downshift of 150 cm^–1^. ^15^NO substitution downshifts the calculated ν_NO_ of neutral and anionic Fe(TPC)(NO) to 1758 and 1619 cm^–1^, again in excellent accord with experiment (1735 to 1597 cm^–1^). The impressive agreement between calculated and experimental ν_NO_ values strongly suggests that spin-unrestricted (broken-symmetry) B3LYP affords an accurate picture of the electronic–structural changes accompanying one-electron reduction of Fe(TPC)(NO). By way of comparison, spin-restricted B3LYP calculations grossly overestimate the ν_NO_ of Fe(TPC)(NO) at 1869.8 cm^–1^ (1830.6 for ^15^NO), which is considerably higher than the experimental value.^21^

**Figure 5:**
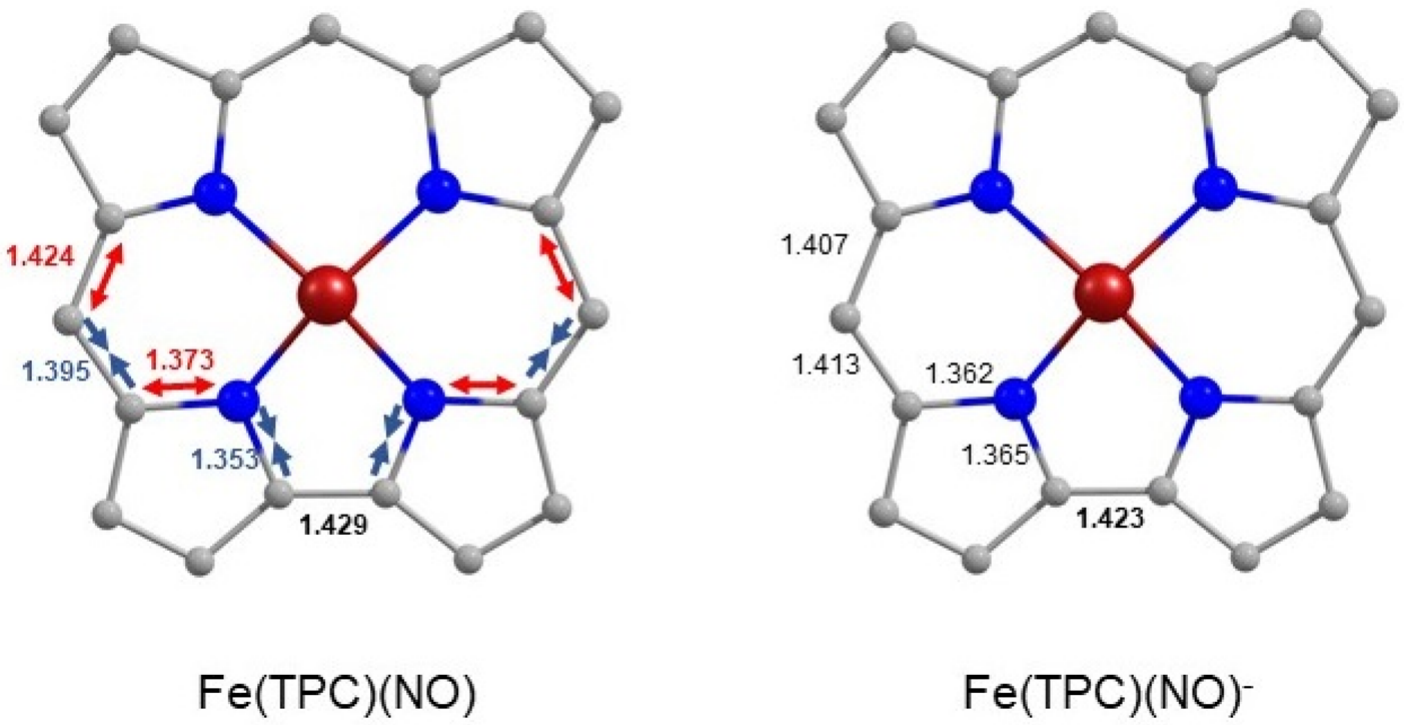
Bond length alternation in Fe(TPC)(NO) (left) as compared to Fe(TPC)(NO)^−^ (right). Bond lengths are in angstroms (Å).

The (broken-symmetry) spin density plots of Fe(TPC)(NO) and Fe(TPC)(NO)^−^ show an almost complete lack of excess spin density on the corrole macrocycle of the anion, suggesting that one-electron reduction neutralizes the corrole radical:
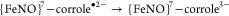


An examination of the corrole skeletal bond distances within and adjacent to the bipyrrole unit also shows that the characteristic bond length alternation observed for the neutral complex has vanished in the anion, consistent with the lack of ligand radical character in the latter species (Figure 5). A detailed examination of Mulliken and NBO charges in the neutral and anionic complexes confirms that the electron adds largely (about 85%) on the corrole, with only about 15% on the NO (Table 4).

**Table 4:** Selected Atomic Charges and Spin Populations from Spin-Unrestricted (Broken-Symmetry) B3LYP/STO-TZP Calculations on Fe(TPC)(NO) and Fe(TPC)(NO)^−^

property	species	Fe	N_NO_	O_NO_	TPC
NBO charge	Fe(TPC)(NO), *M*_S_ = 0	0.941	0.106	–0.218	–0.829
	Fe(TPC)(NO)^−^, *M*_S_ = 1/2	1.060	–0.004	–0.301	–1.755
Mulliken charge	Fe(TPC)(NO), *M*_S_ = 0	0.976	0.023	–0.202	–0.797
	Fe(TPC)(NO)^−^, *M*_S_ = 1/2	0.989	–0.033	–0.275	–1.681
NBO spin pop.	Fe(TPC)(NO), *M*_S_ = 0	1.679	*–0.477*	*–0.467*	*–0.735*
	Fe(TPC)(NO)^−^, *M*_S_ = 1/2	1.946	*–0.572*	*–0.493*	*0.119*
Mulliken spin pop.	Fe(TPC)(NO), *M*_S_ = 0	1.853	*–0.587*	*–0.453*	*–0.814*
	Fe(TPC)(NO)^−^, *M*_S_ = 1/2	2.104	*–0.667*	*–0.477*	*0.041*

**Figure 6:**
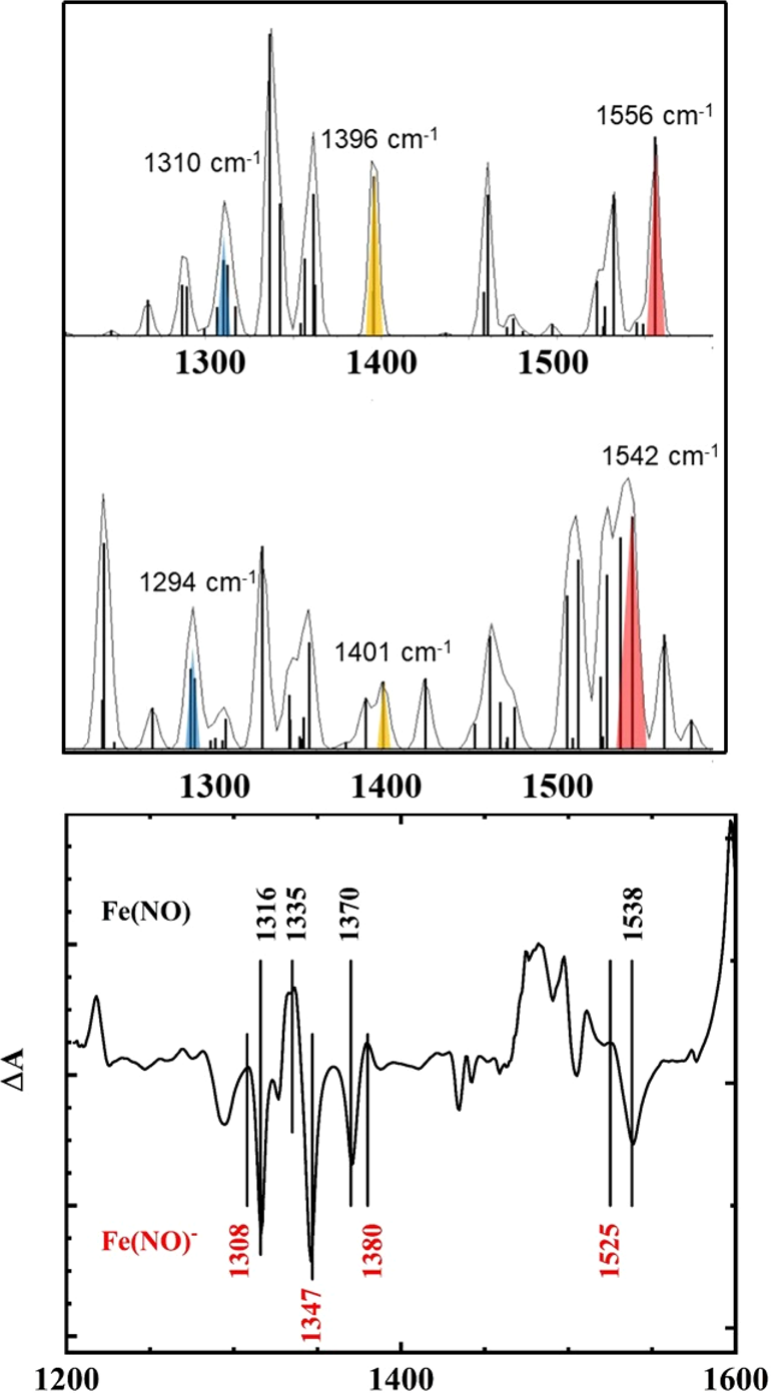
Top spectrum: DFT-simulated infrared spectrum of Fe(TPC)(NO). Middle spectrum: DFT-simulated infrared spectrum of Fe(TPC)(NO)^−^. Selected structure-sensitive bands are highlighted. Bottom spectrum: difference experimental spectrum for the first reduction of Fe(TPC)(NO). Fe(TPC)(NO) bands are labeled in black; Fe(TPC)(NO)^−^ bands are labeled in red.

The calculated infrared spectra for Fe(TPC)(NO) and Fe(TPC)(NO)^−^ are shown in Figure 6, along with the experimental difference spectrum for the reduction of Fe(TPC)(NO) (infrared spectroelectrochemistry of Fe(TPC)(NO) is in Figure S10). The calculated vibrational modes for three of the corrole bands are shown in Figure S11. The spectral changes observed upon reduction were found to correlate well with the calculated DFT spectra. The DFT band at 1550 cm^–1^ for Fe(TPC)(NO) downshifted upon reduction to 1540 cm^–1^. The 1550 cm^–1^ band has been correlated with the 1538 cm^–1^ in the experimental spectrum. The experimental spectrum shows a 13 cm^–1^ downshift to 1525 cm^–1^, along with two additional new bands at lower energy, which can also be seen in the DFT spectrum of Fe(TPC)(NO)^−^. The 1396 cm^–1^ band (1392 cm^–1^ in a previous work^3^), which has been correlated with the 1370 cm^–1^ experimental band,^3^ upshifted to 1401 cm^–1^ in the DFT spectrum (1380 cm^–1^, experimentally). The 1344 cm^–1^ band downshifted to 1330 cm^–1^ (DFT), while the experimental band downshifted from 1347 to 1335 cm^–1^. Finally, the 1310 cm^–1^ DFT band (1316 cm^–1^, exp.) downshifted to 1294 cm^–1^ (1308 cm^–1^, exp.).

The results of the infrared spectroelectrochemistry of the Fe(TPC)(NO) show that the shifts in both the nitrosyl band and the corrole core bands are consistent with reduction occurring primarily on the corrole macrocycle rather than the FeNO moiety.

## Conclusions

The changes in the infrared spectroelectrochemistry of the Fe(corrole)(NO) complexes were consistent with DFT calculations which predicted substantial {FeNO}^7^–corrole^•2–^ character for the neutral complex.^3^ The results confirmed that the corrole is noninnocent in the neutral Fe(corrole)(NO) complex and is the site for the first electron reduction. DFT calculations have shown that the observed downshift in the ν_NO_ band upon reduction reflects in large part the bending of the Fe–N–O moiety as the corrole is reduced. Reduction of the corrole was confirmed by the shifts in the corrole vibrations, which were consistent with the DFT calculations. The results underscore that one must be careful in correlating downshifts in the nitrosyl band with the site of reduction. The use of infrared spectroelectrochemistry along with deuterated solvents provided for a wide spectral window to evaluate the vibrational changes due to reduction. Further reduction to the dianion yielded visible spectral changes that were similar to those accompanying the formation of {FeNO}^8^ porphyrin complexes, for which DFT calculations have shown that the reduction is centered on the NO moiety.^13^ For the second reduction, the changes to the visible spectra were minimal. Further studies are in progress using infrared spectroelectrochemistry to confirm the nature of this process.

## Supporting Information Available

Cyclic voltammetry of Fe(TPC)(NO), visible and infrared spectroelectrochemistry of iron nitrosyl corroles, and visual depiction of key vibrational eigenvectors (PDF)
