# Supplementary material for: Infrared Spectroelectrochemistry of Iron-Nitrosyl Triarylcorroles. Implications for Ligand Noninnocence
Source: Inorg Chem. 2020 Feb 13;59(5):3232–8. doi: 10.1021/acs.inorgchem.9b03613 (PMC7997370; doi:10.1021/acs.inorgchem.9b03613)

Infrared Spectroelectrochemistry of Iron-Nitrosyl Triarylcorroles. Implications for Ligand Noninnocence

Md. Hafizur Rahman [a], Michael D. Ryan [a*], Hugo Vazquez-Lima [b,c], Abraham Alemayehu [b], and Abhik Ghosh [b*]

a. Department of Chemistry, Marquette University, 1414 W Clybourn St., Milwaukee, WI 53233

b. Department of Chemistry, UiT – The Arctic University of Tromsø, 9037 Tromsø, Norway

c. Centro de Química, Instituto de Ciencias, Universidad Autónoma de Puebla, Edif. IC9, CU, San Manuel, 72570 Puebla, Puebla, Mexico.

Table of Contents.

Figure S1. Cyclic voltammetry of 0.5 mM Fe(TPC)(NO) in THF/0.10 M TBAP at a platinum electrode 4

Figure S2. Visible spectroelectrochemistry of the first reduction of 0.48 mM Fe(TPC)(NO) in THF and 0.10 M TBAP. 5

Figure S3. Visible spectroelectrochemistry of the first reduction of 0.43 mM Fe(T*p*CH_3PC)(NO) in THF and 0.10 M TBAP 6

Figure S4. Visible spectroelectrochemistry of the second reduction of 0.48 mM Fe(TPC)(NO) in THF and 0.10 M TBAP 7

Figure S5. Visible spectroelectrochemistry of the second reduction of 0.43 mM Fe(T*p*CH_3PC)(NO) in THF and 0.10 M TBAP 8

Figure S6. Infrared spectroelectrochemistry of the first reduction of 7.0 mM Fe(T*p*CF_3PC)(^{15}NO) in THF-d_8 and 0.10 M TBAP 9

Figure S7. FTIR spectrum of Fe(T*p*CF_3PC)(^{15}NO) in KBr 10

Figure S8. FTIR spectrum of 7.0 mM Fe(T*p*CF_3PC)(^{15}NO) in THF-d_8 after subtraction of THF-d_8/0.10 M TBAP 11

Figure S9. FTIR spectrum of 4.5 mM Fe(T*p*CF_3PC)(^{15}NO)$^-$ in THF-d_8 after subtraction of THF-d_8/0.10 M TBAP 12

Figure S10. Infrared spectroelectrochemistry of the first reduction of 4.5 mM Fe(TPC)(^{14}NO) in THF-d_8 and 0.10 M TBAP....13

Figure S11. Visual Depiction of key structure-sensitive vibrational eigenvectors, along with the corresponding DFT frequencies..14

Figure S1. Cyclic voltammetry of 0.5 mM Fe(TPC)(NO) in THF/0.10 M TBAP at a platinum electrode. Scan rate = 100 mV/s. Uncompensated resistance: 50 k Ω.

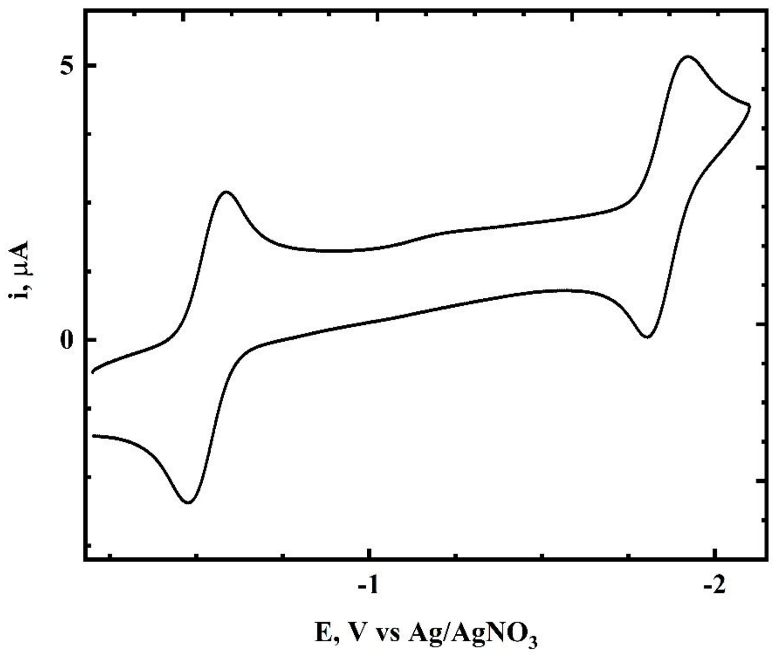

Figure S2. Visible spectroelectrochemistry of the first reduction of 0.48 mM Fe(TPC)(NO) in THF and 0.10 M TBAP. Initial spectrum: -0.400 V (black); intermediate spectra: -0.656V, -0.716 V, -0.776 V, -0.836 V and -0.936 V (green); final spectrum: -1.156 V (red).

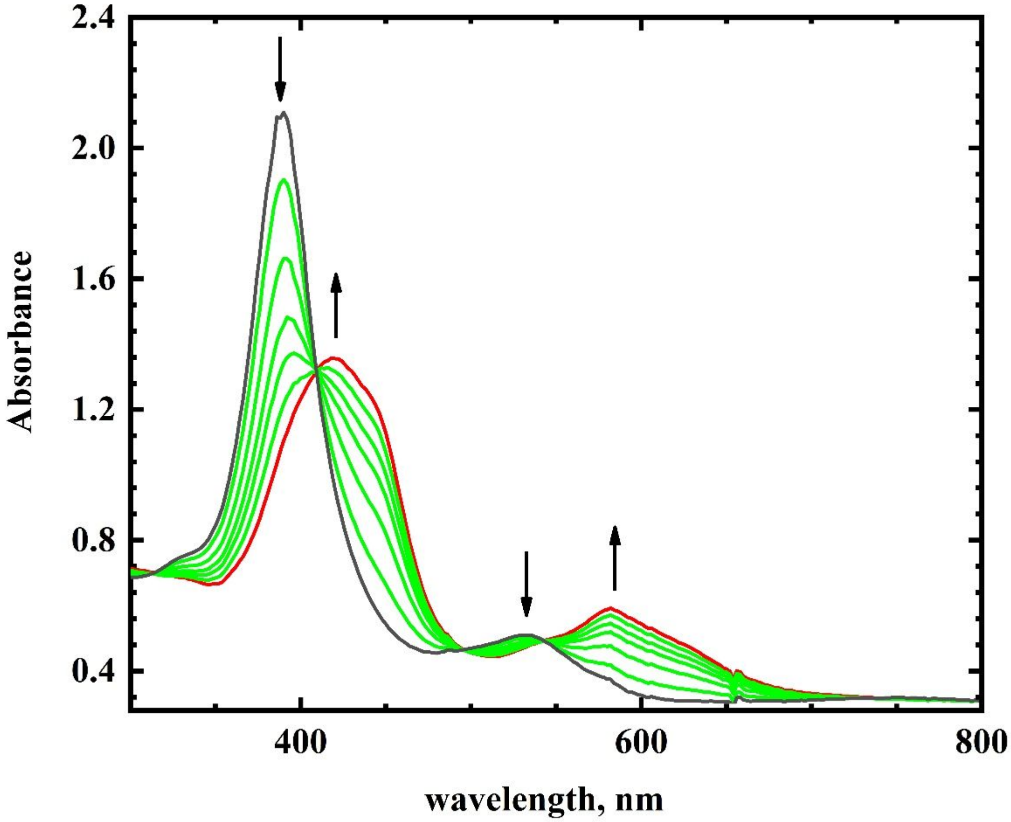

Figure S3. Visible spectroelectrochemistry of the first reduction of 0.43 mM Fe(T*p*CH_3PC)(NO) in THF and 0.10 M TBAP. Initial spectrum: -0.400 V (black); intermediate spectra: -0.694 V, -0.754 V, -0.814 V and -0.934 V (green); final spectrum: -1.264 V (red).

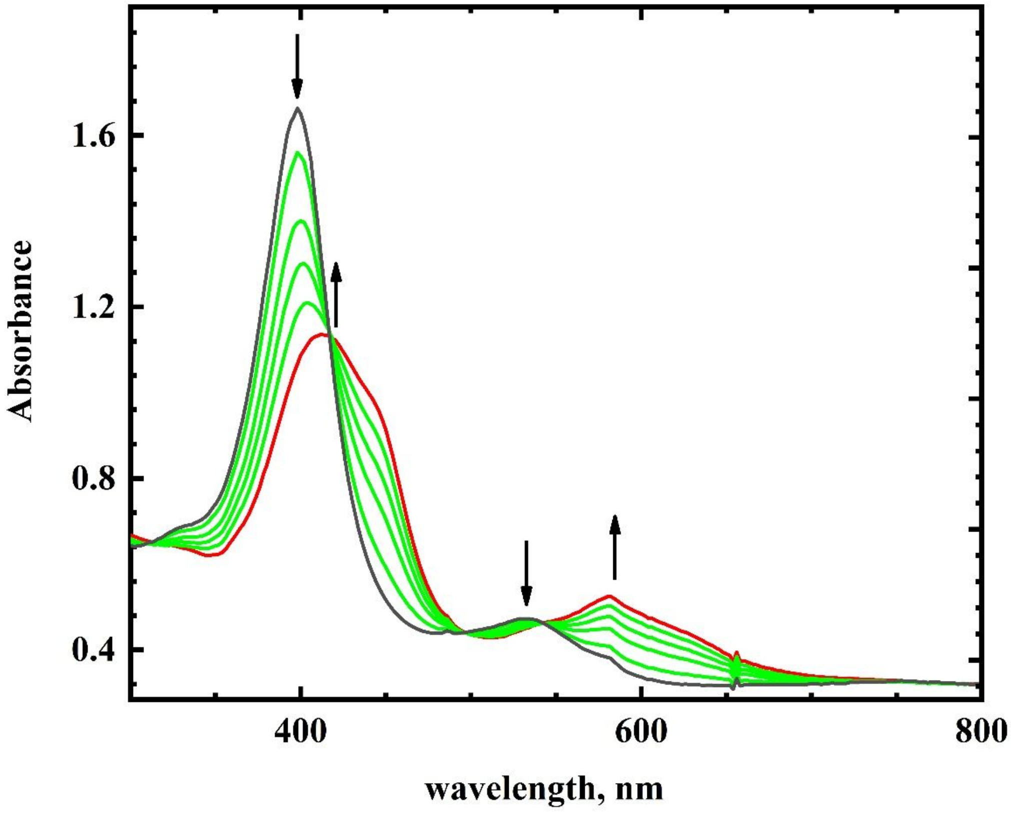

Figure S4. Visible spectroelectrochemistry of the second reduction of 0.48 mM Fe(TPC)(NO) in THF and 0.10 M TBAP. Initial spectrum: -1.676 V (black); intermediate spectra: -1.756 V, -1.876 V, and -1.916 V (green); final spectrum: -2.000 V (red).

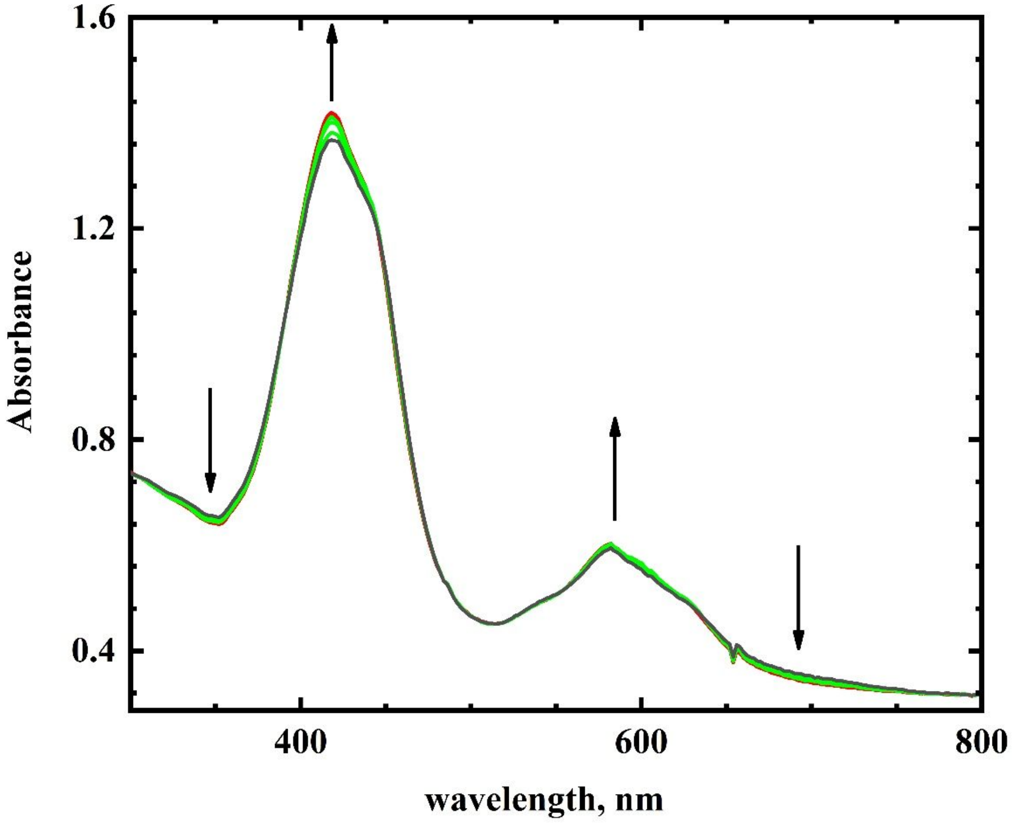

Figure S5. Visible spectroelectrochemistry of the second reduction of 0.43 m<u>M</u> Fe(T*p*CH_3PC)(NO) in THF and 0.10 M TBAP. Initial spectrum: -1.264 V (black); intermediate spectra: -1.366 V, -1.444 V, -1.504 V, -1.586 V (green); final spectrum: -1.654 V (red).

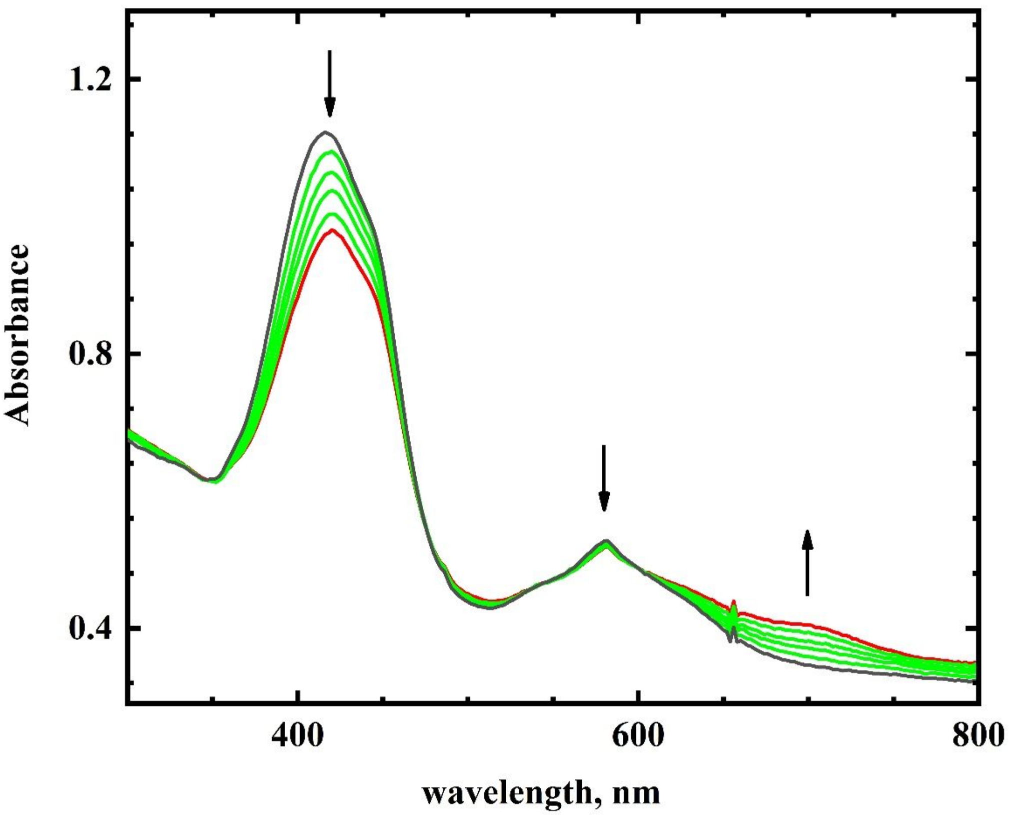

Figure S6. Infrared spectroelectrochemistry of the first reduction of 7.0 mM Fe(T*p*CF_3PC)(^{15}NO) in THF-d_8 and 0.10 M TBAP. Initial spectrum: black; intermediate spectra: green; final spectrum: blue.

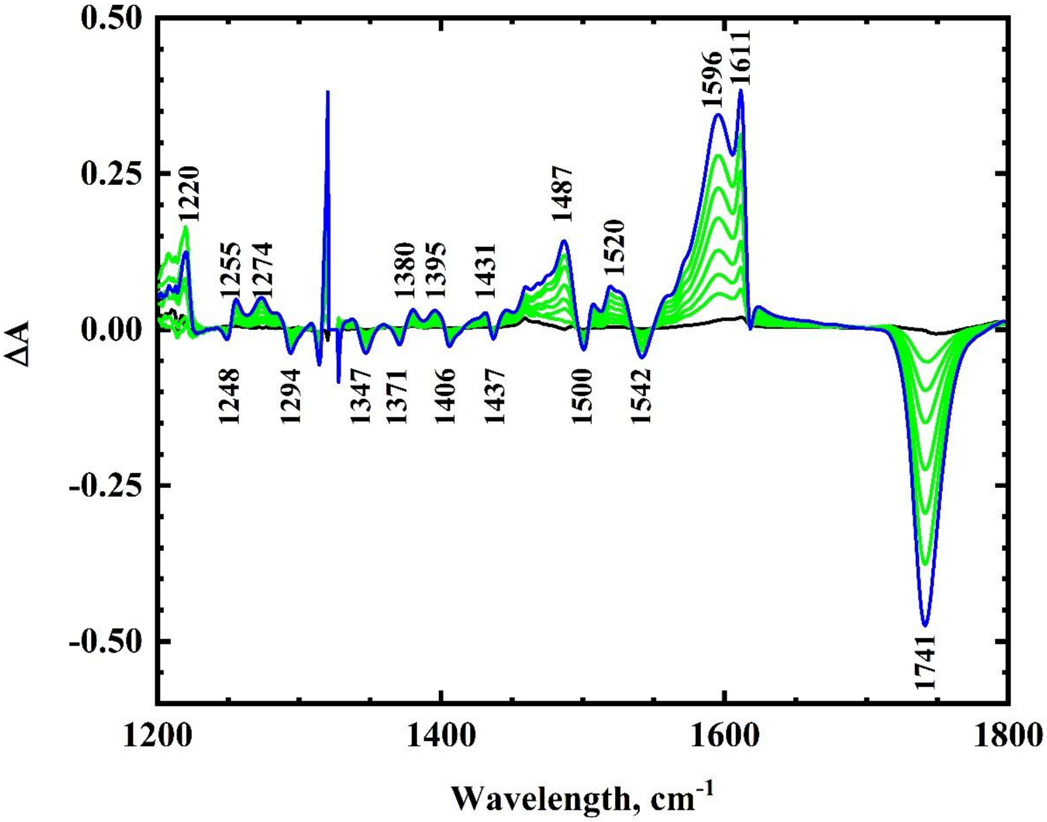

Figure S7. FTIR spectrum of Fe(T*p*CF_3PC)(^{15}NO) in KBr.

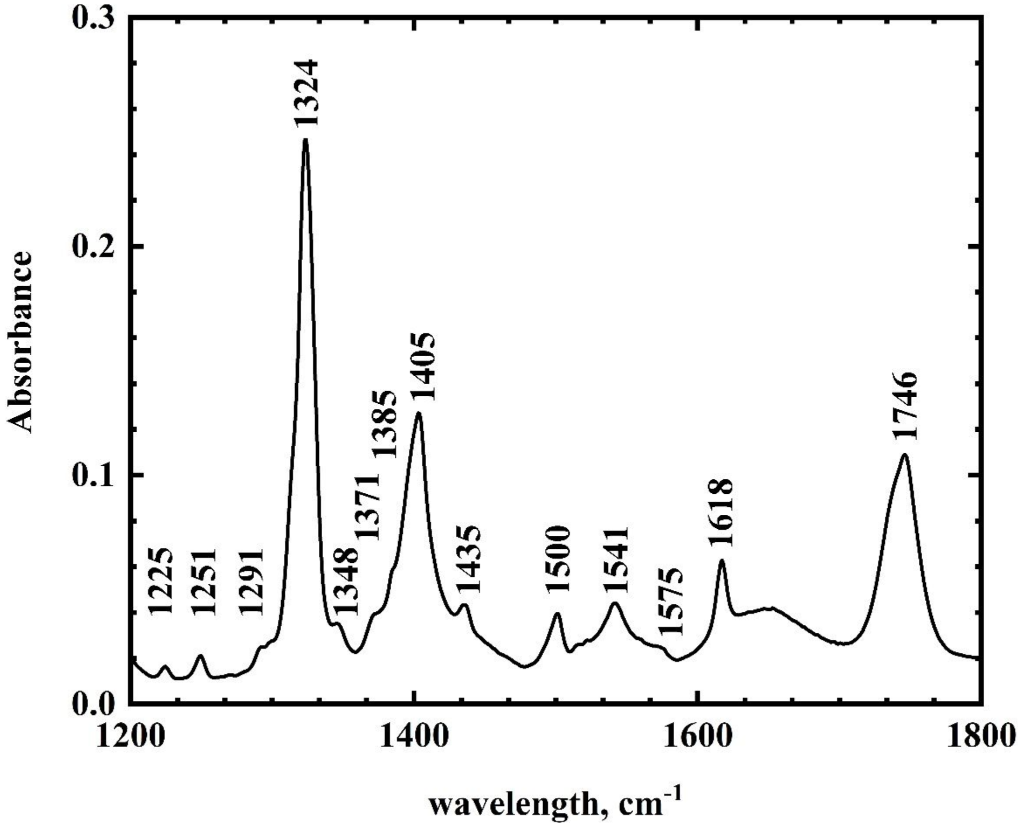

Figure S8. FTIR spectrum of 7.0 m$\underline{M}$ Fe(T*p*CF_3PC)(^{15}NO) in THF-d_8 after subtraction of THF-d_8/0.10 $\underline{M}$ TBAP.

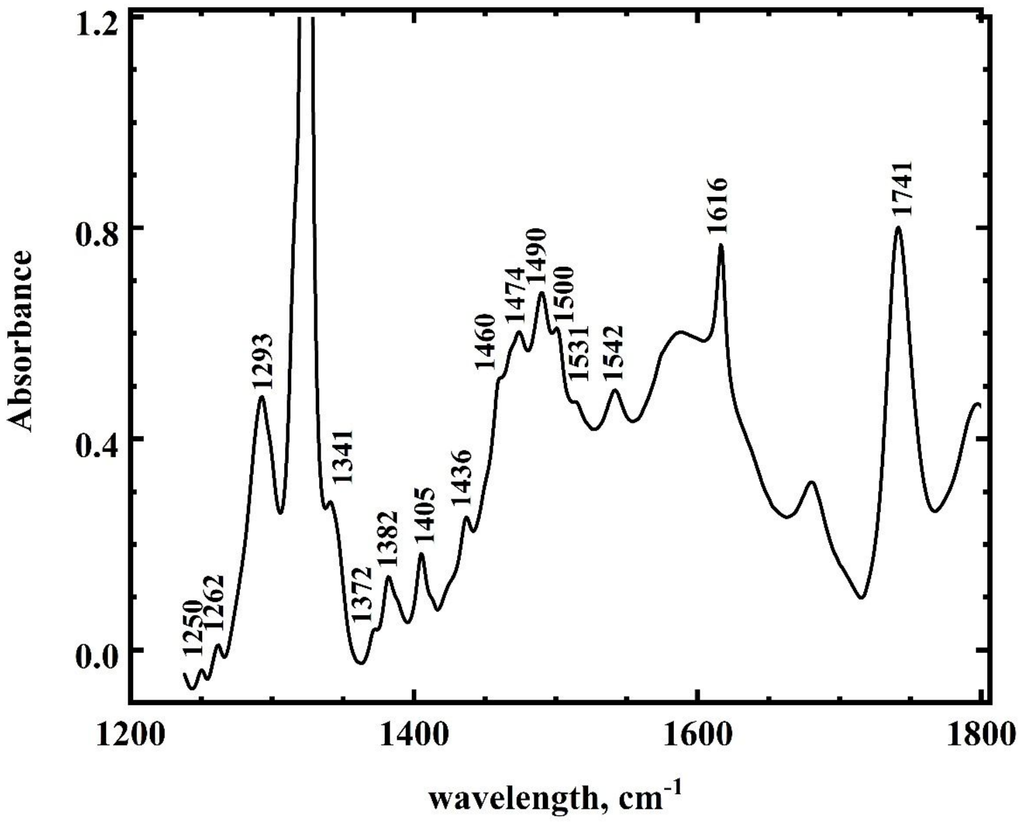

Figure S9. FTIR spectrum of 7.0 m$\underline{M}$ Fe(T*p*CF_3PC)(^{15}NO)$^-$ in THF-d_8 after subtraction of THF-d_8/0.10 $\underline{M}$ TBAP.

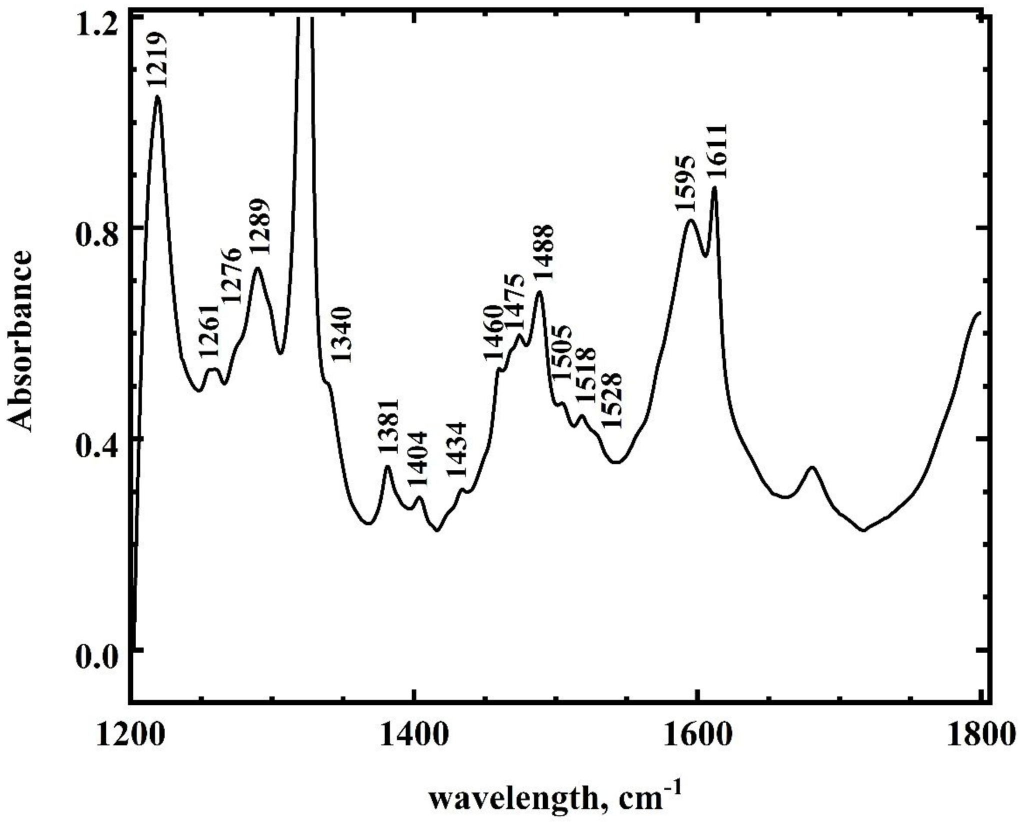

Figure S10. Infrared spectroelectrochemistry of the first reduction of Fe(TPC)(^{14}NO) in THF-d_8 and 0.10 M TBAP.

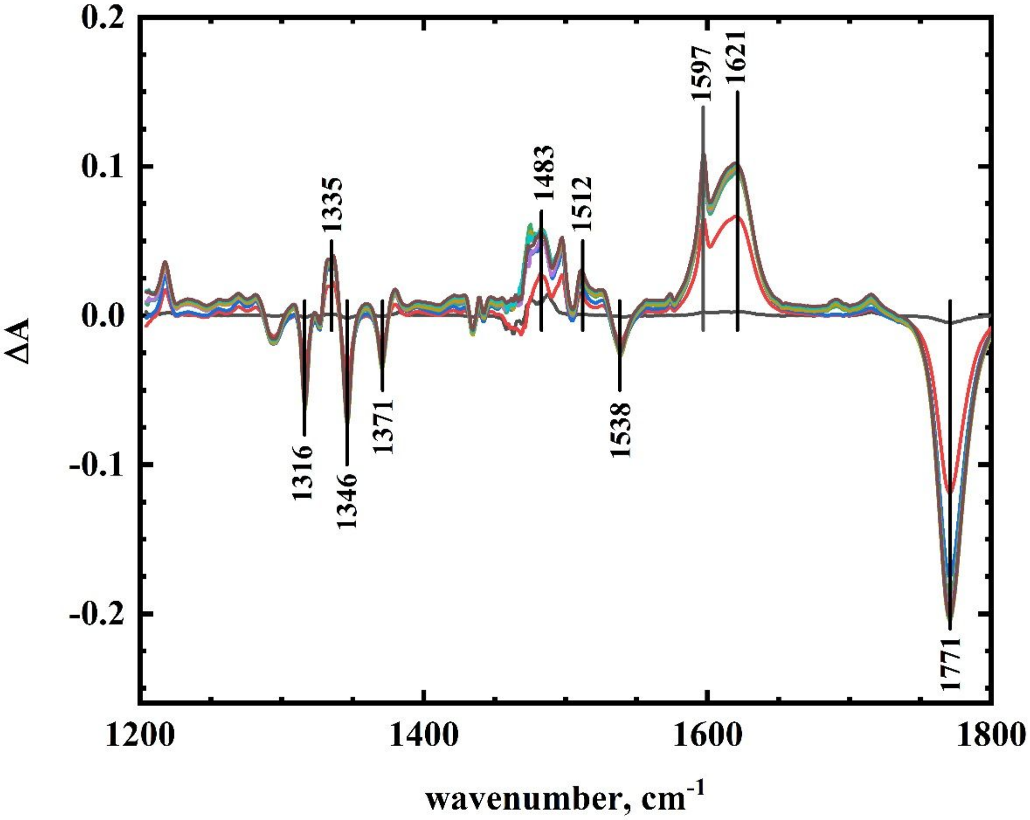

Figure S11. Visual Depiction of key structure-sensitive vibrational eigenvectors, along with the corresponding DFT frequencies. Top set is for Fe(TPC)(NO) and bottom set is for Fe(TPC)(NO)$^{-}$.

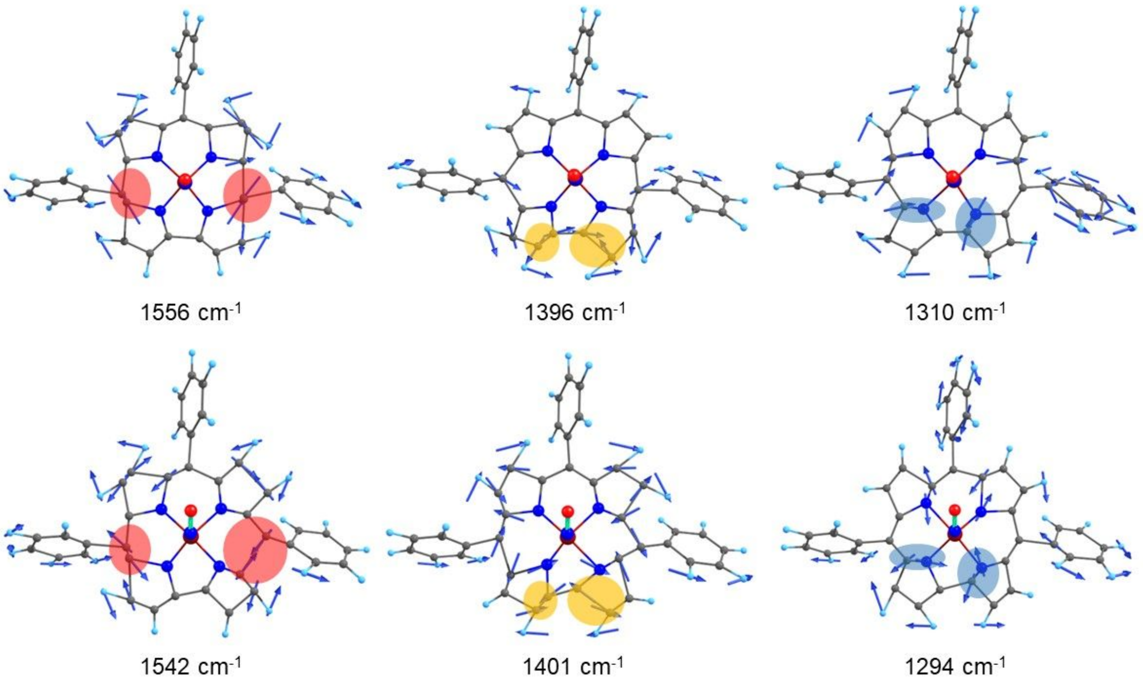

Supplement: Supplementary file 1 — ic9b03613_si_001.pdf [file ic9b03613_si_001.pdf]
